# Modulation of High-Frequency rTMS on Reward Circuitry in Individuals with Nicotine Dependence: A Preliminary fMRI Study

**DOI:** 10.1155/2024/5673579

**Published:** 2024-08-28

**Authors:** Tao Wang, Ruiyang Li, Dongyan Chen, Mei Xie, Zhiqiang Li, Huan Mao, Yuting Ling, Xiaoyun Liang, Guojun Xu, Jianjun Zhang

**Affiliations:** ^1^ Department of Radiology Zhejiang Hospital School of Medicine Zhejiang University, Hangzhou, China; ^2^ Yiruide Medical Equipment New Technology Co. Ltd., Wuhan, China; ^3^ Institute of Research and Clinical Innovations Neusoft Medical Systems Co. Ltd., Shanghai, China; ^4^ Key Laboratory for Biomedical Engineering of Ministry of Education Department of Biomedical Engineering College of Biomedical Engineering and Instrument Science Zhejiang University, Hangzhou, China

## Abstract

Although previous studies have shown that repetitive transcranial magnetic stimulation (rTMS) can ameliorate addictive behaviors and cravings, the underlying neural mechanisms remain unclear. This study aimed to investigate the effect of high-frequency rTMS with the left dorsolateral prefrontal cortex (L-DLPFC) as a target region on smoking addiction in nicotine-dependent individuals by detecting the change of spontaneous brain activity in the reward circuitry. We recruited 17 nicotine-dependence participants, who completed 10 sessions of 10 Hz rTMS over a 2-week period and underwent evaluation of several dependence-related scales, and resting-state fMRI scan before and after the treatment. Functional connectivity (FC) analysis was conducted with reward-related brain regions as seeds, including ventral tegmental area, bilateral nucleus accumbens (NAc), bilateral DLPFC, and bilateral amygdala. We found that, after the treatment, individuals showed reduced nicotine dependence, alleviated tobacco withdrawal symptoms, and diminished smoking cravings. The right NAc showed increased FC with right fusiform gyrus, inferior temporal gyrus (ITG), calcarine fissure and surrounding cortex, superior occipital gyrus (SOG), lingual gyrus, and bilateral cuneus. No significant FC changes were observed in other seed regions. Moreover, the changes in FC between the right NAc and the right ITG as well as SOG before and after rTMS were negatively correlated with changes in smoking scale scores. Our findings suggest that high-frequency L-DLPFC-rTMS reduces nicotine dependence and improves tobacco withdrawal symptoms, and the dysfunctional connectivity in reward circuitry may be the underlying neural mechanism for nicotine addiction and its therapeutic target.

## 1. Introduction

Nicotine, a constituent of tobacco, is one of the most widely abused addictive substances today [1, 2], which can damage the cardiovascular system, leading to increased risks of atherosclerosis, high blood pressure, and blood clot formation, thereby raising the risk of stroke [3]. Nicotine's addictive properties are characterized by an intense craving for acquisition and use, a loss of control over intake, and the development of withdrawal symptoms during cessation efforts [4]. Previous studies [5, 6, 7] have shown that nicotine dependence leads to structural and functional changes in the brain, resulting in cognitive impairments such as decreased attention, working memory, and visual–spatial processing as well as emotional disturbances [5]. However, only 3%–5% of the smoking population succeeds in achieving long-term tobacco cessation through voluntary withdrawal efforts without external intervention [1, 8]. Therefore, nicotine withdrawal treatment often requires external intervention, and the current conventional method for treating tobacco addiction is nicotine replacement therapy [9], which includes substances like chewing gum and oral or nasal inhalers. However, their requirement for frequent administration over time often leads to poor adherence, increased costs, and potential side effects in the nasal, oral, or gastrointestinal tract.

Repetitive transcranial magnetic stimulation (rTMS) is a noninvasive brain stimulation therapy based on the principle of electromagnetic induction [10, 11]. High-frequency rTMS (HF-rTMS) at frequencies of ≥5 Hz enhances neuronal synaptic function, while low-frequency rTMS (LF-rTMS) at frequencies of <1 Hz can inhibit neuronal synaptic activity [12]. Currently, rTMS has been applied to the treatment of drug addiction and various psychiatric disorders [13, 14], showing efficacy in reducing craving associated with stimulus addiction and behavioral addiction cravings [15]. The left dorsolateral prefrontal cortex (L-DLPFC) is a hub region involved in cognitive control and decision-making, and its dysfunction is associated with a decreased ability to resist smoking urges in nicotine-dependent individuals [16]. Studies have indicated that high-frequency TMS stimulation of the L-DLPFC can reduce drug cravings in individuals with substance abuse and improve self-perception and emotional regulation [13, 14, 17, 18], and it is effective in reducing nicotine dependence, increasing tobacco abstinence rates, and improving anxiety and depression-related symptoms [19, 20]. Altered brain functional activity following rTMS can be detected by functional magnetic resonance imaging (fMRI) [21, 22]. In recent years, resting-state fMRI has been extensively utilized to investigate neural mechanisms underlying treatments for substance use disorders (SUDs), including heroin dependency and smoking behavior [23, 24, 25, 26]. For instance, in heroin-dependent individuals, HF-rTMS significantly increased functional connectivity (FC) between the L-DLPFC and the hippocampus, whereas decreased FC between the right precentral gyrus and key areas of the default mode network (DMN) [25]. In nicotine-dependent individuals, HF-L-DLPFC-rTMS resulted in reduced activity in right insula and thalamus, and decreased FC between the L-DLPFC and the left medial orbitofrontal cortex (mOFC) [26]. Additionally, HF-L-DLPFC-rTMS can enhance top-down control over tobacco cravings by strengthening the FC of the frontal–striatal pathway [27].

The reward circuit plays a pivotal role in nicotine addiction, which usually includes the ventral tegmental area (VTA), nucleus accumbens (NAc), prefrontal cortex (PFC), and amygdala (AMYG) [28, 29], involving feelings of reward, pleasure, motivation, and reinforcement learning. Nicotine has an interaction with the neuronal nicotinic acetylcholine receptors (nAChRs) in the VTA and NAc [30, 31], enhancing dopamine transmission, thereby generating rewarding properties that motivate the use of tobacco products. The heightened dopamine activity strengthens the memories of behaviors and experiences associated with nicotine use, which leads to an intensified craving for nicotine, promoting the continuous development of addictive behavior. Moreover, nicotine may also enhance its addictive potential by altering the activity of other neurotransmitter systems, such as glutamate and gamma-aminobutyric acid (GABA) [15, 31]. DLPFC-rTMS may influence the reward circuit system by facilitating an increase in dopamine release and inducing neurotransmitter changes [15, 32]. Previous studies on SUDs observed changes in the FC of reward circuit-related regions [23, 24, 25, 26, 33], such as increased connectivity between the L-DLPFC and dorsal anterior cingulate cortex (dACC) and decreased connectivity between the dACC and mOFC following TMS treatment [33] as well as reduced FC between the L-DLPFC and the mOFC [26], which correlated with improvements in addictive behavior. However, there has been insufficient in-depth analysis of other nodes within the reward circuit [6, 29]. Consequently, the changes in neural circuit function before and after intervention with L-DLPFC-rTMS in nicotine dependence are not yet fully understood [24].

Given the lack of exploration into the FC within the reward network and between reward loop nodes and other brain regions in previous L-DLPFC-rTMS studies [19, 20, 24], this study aimed to assess the changes in clinical addiction characteristics and alterations in the FC of the reward network following rTMS treatment in individuals with nicotine addiction. We also analyzed the correlation between clinical addiction assessments and FC changes. We hypothesized that after HF-L-DLPFC-rTMS treatment, individuals with nicotine addicts would exhibit improvements in clinical addiction characteristics, and their FC in reward circuit-related regions would show changes associated with tobacco addiction.

## 2. Materials and Methods

### 2.1. Participants

**Figure 1:**
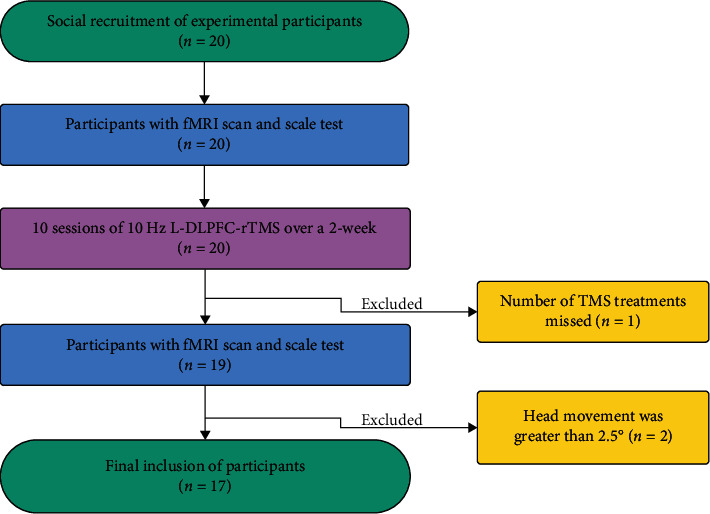
Flow chart for inclusion of participants.

From January 2022 to May 2023, 20 smoking cessation volunteers were recruited through advertisements and online promotions for the study. Inclusion criteria for the nicotine dependence group were as follows: (1) subjects who had ≥10 cigarettes per day for more than 2 years, as defined in the Diagnostic and Statistical Manual of Mental Disorders 5th edition (DSM−5) [34]; (2) male; (3) right-handed; and (4) those who were 18–56 years old. Exclusion criteria were as follows: (1) pregnant and lactating women; (2) those with a history of other brain diseases (e.g., cerebral infarction, cerebral hemorrhage, and brain tumors); (3) those with other organ failure, such as cardiac or pulmonary insufficiency; (4) those who are unable to undergo magnetic resonance examination; (5) those with a history of epilepsy; (6) those with a history of psychotropic drug use within 4 weeks; and (7) those with a history of addiction to other substances. Two subjects were excluded because the head movement was greater than 2.5° during resting-state fMRI scanning, and one subject was excluded because the number of TMS treatments was not up to standard. During the rTMS treatment period, all participants did not receive any additional interventions, such as pharmacological treatment, psychological support, or dietary and exercise interventions. Finally, 17 smoking cessation volunteers were included (Figure 1).

This study was reviewed and approved by the Medical Ethics Committee of Zhejiang Hospital Affiliated with Zhejiang University (2019 Proexamination-34K No.-X2). All subjects were informed of the contents and methods in detail before the examination and voluntarily signed an informed consent form. During the experimental phase, we ensured strict adherence to the World Medical Association's (WMA) Helsinki Declaration of 2013, the Council for International Organizations of Medical Sciences (CIOMS) International Ethical Guidelines for Biomedical Research Involving Human Subjects of 2016, and the ethical review measures outlined by the National Health and Family Planning Commission for Biomedical Research Involving Human Subjects in 2016.

### 2.2. TMS Administration

The rTMS method was built upon prior researches [20, 32]. Ten sessions of TMS (model no. YRD CCY-II) treatment were completed within 2 weeks for each participant. Localization cap positioning with stimulation targeting the L-DLPFC was applied. The stimulation parameters were as follows: 10 Hz frequency at 100% intensity, with continuous stimulation time of 5 s and an interval of 20 s, resulting in a total time of 15 min and a total of 1,500 stimulations.

### 2.3. Clinical Assessment

The clinical characteristics of the patients were assessed before and after 2 weeks of intervention using the Heaviness of Smoking Index (HSI), Fagerström Test for Nicotine Dependence (FTND), Minnesota Tobacco Withdrawal Symptoms Scale (MNWS), and short questionnaire on cigarette craving (QSU-Brief) [35, 36]. The HSI measures the severity of tobacco use; the FTND focuses more on an individual's dependence on nicotine and is the most widely used; the MNWS pays closer attention to the severity of nicotine withdrawal symptoms during cessation, including the intensity of symptoms such as anxiety and depression; the QSU-Brief primarily addresses the intensity and nature of a smoker's craving, including a strong desire for smoking and the anticipated positive effects, as well as relief from tension, unease, and other negative emotions [37].

### 2.4. MRI Acquisition

All MRI scans were performed on 3.0 T Siemens Skyra system, with a 20-channel phased-array head coil. Subjects were instructed to maintain calm breathing, close their eyes, and refrain from moving their heads. Special rubber earplugs and nonmagnetic headphones were worn, and both sides of the head were fixed with sponge cushions to minimize head movement artifacts and noise. The 3D T1 scan parameters were as follows: 176 sagittal slices, echo time (TE) = 2.52 ms, repetition time (TR) = 1,900 ms, 1 mm slice thickness with no gap, matrix size = 256 × 256, flip angle = 9°, 1 × 1 mm^2^ in-plane resolution, and acquisition time = 4 : 26 min. The resting-state fMRI scan parameters were as follows: 33 axial slices, thickness/gap = 3.5/0.5 mm, acquisition matrix = 64 × 64, TR = 2,000 ms, TE = 30 ms, flip angle = 90°, field of view = 200 mm × 200 mm, total volume = 240, and acquisition time = 8 : 08 min.

### 2.5. FC Analysis

Preprocessing of resting-state fMRI data was performed in the SPM12 (https://www.fil.ion.ucl.ac.uk/spm/software/spm12) and RESTplus toolkit (http://www.restfmri.net/forum). The main procedures followed the instructions provided in the RESTplus manual [38], including removing the first 10 time points, slice timing correction, head movement correction (subjects with head movement greater than 2.5 mm or 2.5° were excluded [6, 39]), T1-weighted image segmentation, normalization, smoothing (smoothing kernel with a full-width at half-height of 6 mm), detrend, regression of noisy slant variables (including white matter and cerebrospinal fluid signals and head movement parameters), and filtering (0.01–0.08 Hz). The seed-based approach for studying FC, referencing previous studies, utilized the Montreal Neuroscience Institute (MNI) coordinates of seeds (sphere regions with a radius of 5 mm) as determined by the large-scale data platform Neurosynth (https://neurosynth.org/) [6, 40, 41, 42], including the VTA (*x* = 0, *y* = −15, and *z* = −8), bilateral NAc (right: *x* = 12, *y* = 8, and z = −10; left: *x* = −10, *y* = 6, and*z* = −10), bilateral DLPFC (*x* = ±45, *y* = 32, and *z* = 30), and bilateral AMYG (*x* = ±21, *y* = −3, and *z* = −18). Then, we computed the FC among the seeds, and between them and other voxels in the whole brain, before and after rTMS treatment. Finally, the Fisher *z*-transformation was performed for the FC values.

### 2.6. Statistical Analysis

General and clinical data were analyzed using paired *t*-tests and nonparametric rank sum tests, for normally distributed data and nonnormally distributed data, respectively. The FC before and after the treatment were compared using paired samples *t*-tests, with age and years of education as covariates. Statistically significant differences were indicated when the voxel level was *P*  < 0.005 and the cluster level was *P*  < 0.05 (GRF-corrected) for voxel-wise results and FDR-corrected *P*  < 0.05 for those among seeds. Brain regions that exhibited significant FC differences before and after rTMS treatment were further used to analyze the correlation between FC and various tobacco addiction behavioral scales.

## 3. Results

### 3.1. General Information and Clinical Characteristics

FTND, HSI, MNWS, and QSU-Brief of nicotine-dependent individuals before and after rTMS treatment were statistically significant (Table 1).

### 3.2. Functional Connectivity

**Figure 2:**
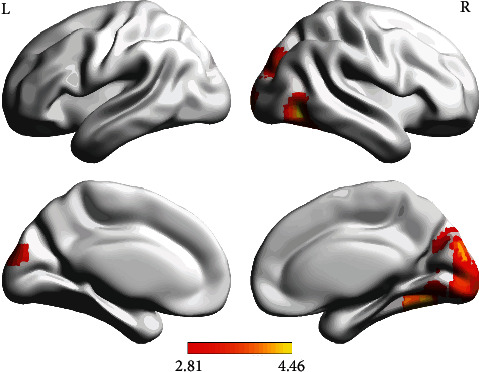
Enhanced functional connectivity between the NAc.R and other regions in nicotine-dependent individuals after the treatment. The color bar represents *t* value (*P*  < 0.05, GRF corrected). NAc.R, right nucleus accumbens.

We found enhanced FC between the right NAc and the right fusiform gyrus (FFG.R), right inferior temporal gyrus (ITG.R), right calcarine fissure and surrounding cortex (CAL.R), right superior occipital gyrus (SOG.R), right cuneus (CUN.R), left cuneus (CUN.L), and right lingual gyrus (LING.R) in nicotine-dependent individuals after the treatment (Table 2 and Figure 2). No significant differences before and after the treatment were observed in the FC of other seeds and among the seeds.

### 3.3. Correlation between FC and Clinical Characteristics

**Figure 3:**
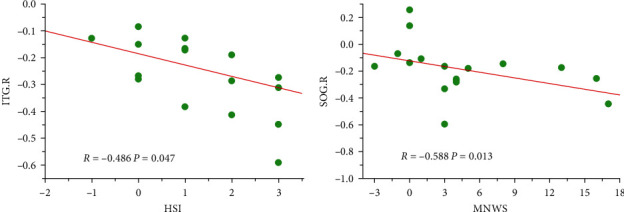
The changes in FC between NAc.R and ITG.R before and after the treatment were negatively correlated with changes in HSI scores (a) and the changes in FC between NAc.R and SOG.R were negatively correlated with changes in MNWS scores (b). ITG.R, right inferior temporal gyrus; SOG.R, right superior occipital gyrus; HSI, Heaviness of Smoking Index; and MNWS, Minnesota Tobacco Withdrawal Symptoms Scale.

The changes in FC between NAc.R and ITG.R before and after the L-DLPFC-rTMS treatment were negatively correlated with changes in HSI scores (*P*=0.047, uncorrected). Similarly, the changes in FC between NAc.R and SOG.R were negatively correlated with changes in MNWS scores (*P*=0.013, uncorrected), as shown in Figure 3.

## 4. Discussion

This study focused on the effects of L-DLPFC-rTMS treatment on the reward circuitry in nicotine addiction. The results supported our hypothesis, and found that (1) HF-DLPFC-rTMS treatment significantly improved nicotine addiction symptoms, as measured by a composite nicotine addiction score; (2) the FC between NAc.R and visual/sensory brain regions showed significant increase after rTMS; and (3) the FC changes negatively correlated with addiction scores. These results highlight the specific neural connectivity alterations associated with L-DLPFC-rTMS treatment in nicotine-dependent individuals, suggesting potential targets for therapeutic interventions.

At present, assessing tobacco addiction behaviors and dependency through scales is a mainstream approach [43, 44]. These scales each have their focus and can collectively reflect an individual's addiction to smoking, though they do not eliminate the shortcomings of strong subjectivity [32]. Our study revealed that 10 sessions of HF-L-DLPFC-rTMS treatment effectively reduced nicotine dependence (HSI and FTND), alleviated tobacco withdrawal symptoms (MNWS), and diminished smoking cravings (QSU-Brief) in nicotine-dependent individuals, aligning with previous findings [20] on L-DLPFC-rTMS treatments for tobacco addiction. These behavioral improvements suggest that L-DLPFC-rTMS may influence the underlying neural mechanisms of tobacco addiction, potentially altering the reward circuit and associated neural interactions. The observed improvements in addiction-related behaviors and symptoms highlight the promise of L-DLPFC-rTMS as a noninvasive treatment option for nicotine dependence. Moreover, although we have not specifically studied differences in rTMS treatment frequency and session duration, current literature suggests that varying frequencies of rTMS have different influences on the treatment efficacy in nicotine addiction [32]. Higher frequencies are generally associated with stronger activation effects, which may arise from stronger neural modulation in the targeted brain areas [45]. However, the higher the frequency and intensity of rTMS, the shorter the duration of stimulation needs to be for safety reasons [46]. The optimal frequency for rTMS treatment of nicotine addiction has not yet been determined and might, therefore, differ among individuals [12]. Further research could explore the long-term effects and optimal protocols for L-DLPFC-rTMS, as well as its application to other forms of substance dependence [10, 24, 47]. Furthermore, no adverse events were observed among participants during rTMS treatment period in this study. Although the literature reports that rTMS might cause mild and transient side effects, such as slight headaches or scalp discomfort, in general, these side effects are only temporary and will disappear once the stimulation stops [46]. This furthermore reflects the safety of L-DLPFC-rTMS treatment.

Prior studies found that both the connectivity within the reward circuit's nodes [6] and its FC with other brain regions, such as striato-cortical circuits [48, 49], exhibited significant abnormalities in nicotine addiction patients. Moreover, the FC patterns within the reward circuit correlated with the severity of nicotine addiction [50], which also led to abnormal connectivity patterns in the executive control and default networks [51], as well as increased connectivity between the NAc and various subcortical areas involved in reward processing [52]. Some studies on L-DLPFC-rTMS treatment for substance addiction have found changes in FC between the DLPFC and other reward-related regions, such as the NAc, caudate nucleus, insula, and DMN-related brain regions [27, 53, 54]. Similar to these studies, L-DLPFC-rTMS effectively reduced smoking cravings and nicotine dependence and altered FC in specific brain regions, particularly those related to the DMN [25, 53]. Studies have reported that the functional impairment of the DMN in smokers may be related to decreased cognitive control and emotional regulation, making it more difficult for them to resist smoking urges and cravings [53, 55]. In our study, DLPFC-rTMS treatment may have improved communication between the reward system and the DMN by enhancing the functional connectivity between NAc and DMN [56]. However, our study did not observe a reduction in FC between L-DLPFC and mOFC in smokers following DLPFC-rTMS treatment [26], which differs from previous findings. We hypothesize that this discrepancy might be related to the remote network effects of rTMS [57], the complex neural mechanisms underlying addiction [54], and the differences in research design and sample characteristics.

Our study primarily highlighted the enhanced FC between NAc.R and several visual and sensory processing regions following L-DLPFC-rTMS treatment. This suggests a reorganization or strengthening of the neural pathways between NAc.R and these brain regions, reflecting increased synchronization of neuronal activity and improved cooperation between these areas [58]. This finding indicates that L-DLPFC-rTMS affects a broader brain network [54], particularly in sensory processing and integration, rather than being limited to changes in connectivity within the reward circuit and executive control network [24, 54]. The FFG is a major brain region involved in memory processing and encoding [59]. The CUN is an important part of DMN [60], which is involved in self-referential thinking, memory, and insight; and the CAL.R, SOG.R, and LING.R are primarily located in the occipital network [56] and related to visual memory and cognitive functions (studies have shown that long-term smoking affects the visuospatial attention system [61, 62, 63]). Therefore, L-DLPFC-rTMS might induce adaptive reorganization in brain networks during the cessation of smoking, especially among regions related to reward, visual memory, cognitive control, and self-emotional regulation, with the NAc.R appearing to play a central role in this process.

We also found that the changes in FC between NAc.R between ITG.R and SOG.R before and after L-DLPFC-rTMS treatment were negatively correlated with changes in smoking scale scores. This finding is consistent with previous studies on L-DLPFC-rTMS treatment for nicotine addiction, where FC changes were also correlated with smoking scores [26, 27, 33]. This indicates that enhanced functional connectivity is associated with reduced smoking behavior, decreased dependence, and alleviation of withdrawal symptoms. Enhanced FC may be one of the neural bases for the therapeutic effects of rTMS [64]. By modulating relevant neural circuits, L-DLPFC-rTMS achieves control over smoking behavior. These findings further suggest that these regions could be potential targets for future research and treatment.

This study has several limitations. First, the remainder of the reward circuit nodes did not exhibit significant FC alterations, failing to meet the anticipated outcomes [6, 48, 49]. Our sample size was relatively small, which may not be reflective of the broader nicotine-dependent population. While studies indicate that L-DLPFC-rTMS has significant short-term effects on smoking cessation, its long-term efficacy is less promising [32, 65], suggesting the need for further research with larger sample sizes and extended follow-up periods to clarify these findings. Additionally, the study lacked a double-blind and sham-controlled design, and the long-term abstinence effects remain unclear. Subjective bias and personal preferences of the experimenter and participants may have slightly affected the results. Some effects of non-rTMS treatments, including placebo and withdrawal effects, were not ruled out [66]. Furthermore, our study participants were all male, and some studies have reported gender differences in nicotine dependence, indicating that the FC results of L-DLPFC-rTMS in female smokers may differ [67]. In future research, we plan to include a sham stimulation control group and incorporate female participants to address these issues.

## 5. Conclusions

HF-L-DLPFC-rTMS can alleviate nicotine dependence and improve tobacco withdrawal symptoms. The enhanced FC between NAc.R and several visual and sensory processing brain regions may play a key role in regulating addictive behaviors. The unaltered functional connectivity patterns within the reward circuit posttreatment may constitute one of the neural mechanisms impeding long-term smoking cessation. Notably, NAc.R within the reward circuit might emerge as a novel potential therapeutic target.

## Figures and Tables

**Table 1:** General information and clinical characteristics of nicotine-dependent individuals before and after the treatment.

Participant characteristics	Pretreatment (*n* = 17)	Posttreatment (*n* = 17)	*P*
Age (year)	38.00 ± 9.45	—	—
Education (year)	13.06 ± 4.05	—	—
Smoking age (year)	17.35 ± 8.19	—	—
Head movement (FD, mm)	0.07 ± 0.04	0.08 ± 0.05	0.266^b^
FTND	3.76 ± 2.14	1.82 ± 1.70	0.002^b^
HSI	2.65 ± 1.58	1.24 ± 1.35	0.003^b^
MNWS	9.47 ± 8.06	4.94 ± 3.90	0.005^a^
QSU-Brief	50.94 ± 16.20	33.35 ± 16.49	<0.001^a^

Data were presented as mean ± SD. ^a^Paired *t*-test; ^b^Wilcoxon signed rank test. FD, frame-wise displacement; HSI, Heaviness of Smoking Index; FTND, Fagerström Test for Nicotine Dependence; QSU-Brief, questionnaire of smoking urges brief; and MNWS, Minnesota Tobacco Withdrawal Symptoms Scale.

**Table 2:** Altered FC between the right NAc and other brain regions before and after the treatment.

Seed region	Brain area	MNI coordinates of peak point (*X*, *Y*, and *Z*)	Number of voxel	*t* value
NAc.R	FFG.R	29, −63, −15	73	4.25
	ITG.R	42, −63, −6	41	4.46
	CAL.R	18, −89, 2	94	3.95
	SOG.R	25, −80, 26	58	3.41
	CUN.R	16, −92, 9	42	2.99
	CUN.L	−6, −94, 21	46	3.43
	LING.R	15, −91, −6	15	3.15

All corrected for GRF, *P*  < 0.05 for voxel level and *P*  < 0.05 for cluster level. MNI, Montreal Neuroscience Institute. NAc.R, right nucleus accumbens; FFG.R, right fusiform gyrus; ITG.R, right Inferior temporal gyrus; CAL.R, right calcarine fissure and surrounding cortex; SOG.R, right superior occipital gyrus; CUN.R, right cuneus; CUN.L, left cuneus; and LING.R, right lingual gyrus.

## Data Availability

The data that support the findings of this study are available from the corresponding author upon reasonable request.
